# Testing the leadership and organizational change for implementation (LOCI) intervention in substance abuse treatment: a cluster randomized trial study protocol

**DOI:** 10.1186/s13012-017-0562-3

**Published:** 2017-03-03

**Authors:** Gregory A. Aarons, Mark G. Ehrhart, Joanna C. Moullin, Elisa M. Torres, Amy E. Green

**Affiliations:** 1 Department of Psychiatry, University of California, San Diego, 9500 Gilman Drive (0812), La Jolla, San Diego, CA 92093-0812 USA; 2 Child and Adolescent Services Research Center, 3665 Kearny Villa Rd., Suite 200N, San Diego, CA 92123 USA; 3 Department of Psychology, San Diego State University, 5500 Campanile Drive, San Diego, CA 92182-4611 USA

**Keywords:** Implementation, Implementation strategy, Leadership, Organizational climate, Attitudes, Fidelity, Motivational interviewing

## Abstract

**Background:**

Evidence-based practice (EBP) implementation represents a strategic change in organizations that requires effective leadership and alignment of leadership and organizational support across organizational levels. As such, there is a need for combining leadership development with organizational strategies to support organizational climate conducive to EBP implementation. The leadership and organizational change for implementation (LOCI) intervention includes leadership training for workgroup leaders, ongoing implementation leadership coaching, 360° assessment, and strategic planning with top and middle management regarding how they can support workgroup leaders in developing a positive EBP implementation climate.

**Methods:**

This test of the LOCI intervention will take place in conjunction with the implementation of motivational interviewing (MI) in 60 substance use disorder treatment programs in California, USA. Participants will include agency executives, 60 program leaders, and approximately 360 treatment staff. LOCI will be tested using a multiple cohort, cluster randomized trial that randomizes workgroups (i.e., programs) within agency to either LOCI or a webinar leadership training control condition in three consecutive cohorts. The LOCI intervention is 12 months, and the webinar control intervention takes place in months 1, 5, and 8, for each cohort. Web-based surveys of staff and supervisors will be used to collect data on leadership, implementation climate, provider attitudes, and citizenship. Audio recordings of counseling sessions will be coded for MI fidelity. The unit of analysis will be the workgroup, randomized by site within agency and with care taken that co-located workgroups are assigned to the same condition to avoid contamination. Hierarchical linear modeling (HLM) will be used to analyze the data to account for the nested data structure.

**Discussion:**

LOCI has been developed to be a feasible and effective approach for organizations to create a positive climate and fertile context for EBP implementation. The approach seeks to cultivate and sustain both effective general and implementation leadership as well as organizational strategies and support that will remain after the study has ended. Development of a positive implementation climate for MI should result in more positive service provider attitudes and behaviors related to the use of MI and, ultimately, higher fidelity in the use of MI.

**Trial registration:**

This study is registered with Clinicaltrials.gov (NCT03042832), 2 February 2017, retrospectively registered.

**Electronic supplementary material:**

The online version of this article (doi:10.1186/s13012-017-0562-3) contains supplementary material, which is available to authorized users.

## Background

Evidence-based practice (EBP) implementation represents a strategic change in organizations that requires effective leadership and the alignment of leadership and organizational support across organizational levels [1, 2]. Although there are many leadership development approaches, few are based on evidence of effectiveness, and to our knowledge, none are specifically designed to develop strategic climates for EBP implementation [3]. Changing the organizational context to support EBP is a critical challenge facing health and allied healthcare settings [4–8]. Leadership is a critical factor in developing an implementation climate [9] and improving attitudes [10–13] toward EBP, as well as clinical outcomes such as client satisfaction and quality of life [14].

**Fig. 1:**
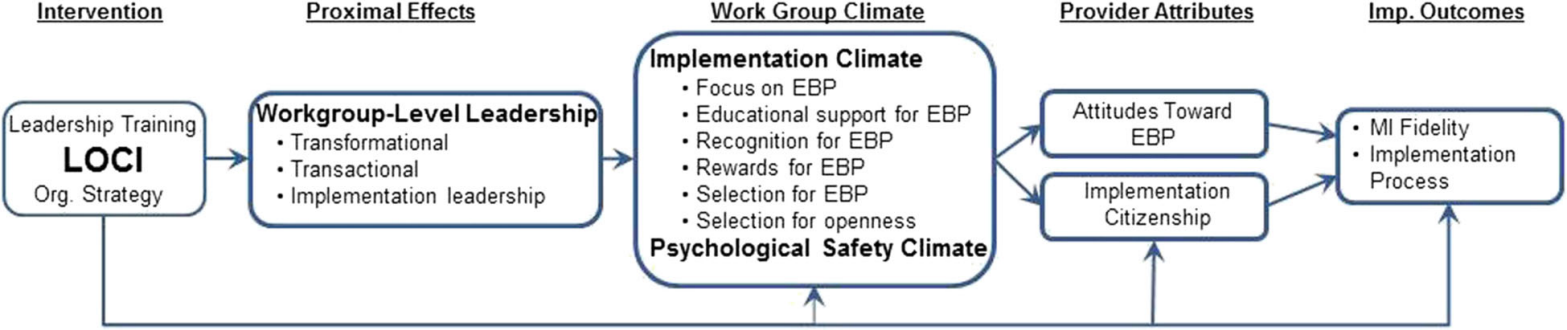
Effects of LOCI on leadership, implementation and psychological safety climate, provider attitudes and citizenship, and implementation outcomes. We will compare LOCI vs. control on proximal and distal outcomes. Exploratory analyses will examine mediational and cross-level effects

Effective EBP implementation to address complex and widespread public health issues, such as substance use disorders (SUDs), remains a significant challenge. Our ability to effectively implement EBPs is as important as the EBP itself because implementation efforts often fail to effectively institute innovations in organizations [15–17]. Although there are process models to facilitate EBP intervention development and implementation [18–20], there are few empirically tested organizational strategies to facilitate EBP implementation in substance use disorder treatment (SUDT) [5, 20–22]. Rigorous testing of implementation strategies for SUDs is in its infancy, particularly for organizationally focused interventions. This study addresses these concerns and advances implementation science by testing the leadership and organizational change for implementation (LOCI) (pronounced lō - sī) intervention. The goals of LOCI are to improve general leadership and implementation leadership combined with the development and use of organizational strategies to create a positive strategic organizational climate to support EBP implementation and sustainment. As shown in Fig. 1, improved leadership, in combination with targeted and multilevel organizational strategies, is hypothesized to lead to improved leadership, implementation climate, and SUDT provider attitudes and behaviors that support the implementation process and implementation outcomes.

This study draws on two leadership approaches, the full-range leadership (FRL) model and implementation leadership [23]. The FRL model is a validated approach to leadership for individual and organizational development [24, 25]. The FRL model includes transformational and transactional leadership and is measured with the reliable and valid Multifactor Leadership Questionnaire [26, 27]. Transformational leadership inspires and motivates employees to follow an ideal or course of action. Transactional leadership reflects a leader’s ability to manage and motivate staff through appropriate interactions and rewards [28]. Appropriate application of the FRL forms the foundation of effective leadership and may impact how employees accept the vision of the leader and follow through on job roles and tasks.

Implementation leadership involves behaviors that fall on four dimensions of being knowledgeable about the EBP being implemented, being proactive and anticipatory in problem-solving, supporting others in the implementation process, and persevering through the ups and downs of the implementation process [23]. The Implementation Leadership Scale assessing these dimensions has been validated for use in SUDT [29]. LOCI utilizes both FRL and implementation leadership as complementary leader skills and behaviors that can be utilized to help to develop a positive EBP implementation climate [13, 30, 31]. Thus, this project advances the application of leadership theory in implementation science.

In contrast to general organizational climate that supports the overall well-being of employees, a strategic climate supports a particular organizational purpose or goal [32, 33]. Implementation climate is a strategic climate defined as “employees’ shared perceptions of the importance of innovation implementation within the organization” [34]. Cross-level relationships between executive management, mid-management, and first-level leadership develop and support congruence of EBP support structures and processes, in a targeted and concerted strategy to improve implementation climate [1, 31].

### LOCI development

LOCI is framed within the Exploration, Preparation, Implementation, Sustainment (EPIS) framework [21], focusing primarily on the inner organizational context and the Preparation and Implementation phases. As shown in Fig. 1, LOCI takes an active approach to improving leadership and congruent organizational strategies that lead to improved transformational and transactional leadership, implementation leadership, and subsequent implementation climate and psychological safety climate. These, in turn, are hypothesized to lead to changes in provider attitudes toward EBP, implementation citizenship behaviors, and to better EBP fidelity and implementation process [4, 35–38]. Consistent with the EPIS conceptual framework, LOCI creates change at multiple levels within a provider organization (e.g., executives/mid-managers, workgroup supervisor, service provider) to foster a context supportive of EBP implementation and sustainment.

It is important to address leadership at the appropriate organizational levels. “First-level” leaders manage and supervise those providing direct services to clients and can be particularly important in influencing staff perceptions and behavior. Workgroup supervisors are first-level leaders and are important in effective workgroup functioning [39], yet public sector first-level leaders are often promoted from clinical or service positions without adequate training in leadership [13]. SUDT workgroup supervisors are frequently responsible for implementing innovations and meeting administrative and productivity requirements [40] but can be organizational “change agents” to inspire and motivate followers to implement change [41, 42]. They play a critical role in staff perceptions of support for using an innovation such as EBP [43]. Clinical workgroup supervisors are likely to be more effective with the buy-in and support of middle and upper organizational management [44–47]. It is important to consider multiple levels within provider organizations when working to create a context supportive of EBP implementation and sustainment [21, 48–50]. LOCI entails developing organizational strategies tailored to support first-level leaders while facilitating buy-in and support from upper and mid-level managements for the strategic goal of effective implementation through the creation of a positive EBP implementation climate. In LOCI, members of the research team support workgroup supervisors and management to develop a set of strategies to embed an EBP implementation climate and support EBP implementation with fidelity [2, 51].

### The evidence-based practice being implemented

Motivational interviewing (MI) is a leading EBP for SUDT. MI was developed after a series of findings illustrated that therapist empathy during treatment, in contrast to the treatment intervention itself, accounted for a larger proportion of variance in SUDT outcomes including post-treatment relapse [52]. MI emphasizes using an empathic client-centered clinical style while evoking and strengthening the client’s own verbalized motivations for overcoming ambivalence about change [53]. In the spirit of MI, it is the patient’s role, as opposed to the clinician’s, to articulate and resolve their own ambivalence regarding change. Treatment providers achieving high levels of MI fidelity are informative, supportive, respectful, and collaborative; their patients are more satisfied, more likely to be retained in care, more committed to treatment regimens, and have better outcomes when compared to those whose providers do not use MI [54–64].

### Preliminary studies

With NIMH support (Grant No. R21MH082731), experts in leadership and implementation from business and management schools were engaged to work with the investigators and mental health program managers (i.e., first-level leaders) and an instructional design consultant to develop the training strategy and materials. Next, a pilot study involving 12 mental health program managers, randomly assigned to LOCI or a leadership webinar, was conducted. Results demonstrated feasibility, acceptability, perceived utility, and change in leadership behavior [1, 23, 65].

### Significance

There are two main potential impacts of the proposed study. First, improving leadership and organizational climate for EBP will advance both implementation science and will provide an empirically tested implementation strategy to decrease the lag between intervention or innovation development and deployment and sustainment in usual care settings [66]. Demonstrating the efficacy of LOCI will provide service systems and health care organizations with a way to simultaneously improve leadership and institute organizational strategies to support and promote effective EBP implementation and sustainment. Second, although counselors and clinicians may receive training in MI, leaders and organizations often do not apply strategies to effectively implement MI or support staff to deliver MI with fidelity beyond initial training [67, 68].

To our knowledge, there are no other empirically tested workgroup level approaches that bring together leadership and organizational strategies to improve strategic climate for EBP and implementation effectiveness. In addition, this proposed study utilizes new reliable and valid measures of implementation leadership [23], implementation climate [30], and implementation citizenship [69] and will apply the Stages of Implementation Completion (SIC) measure [37] to assess LOCI and MI implementation process.

## Methods

This study will test the effects of LOCI in facilitating MI implementation in SUDT service settings.

The study’s aims and hypotheses are:

- Aim 1: Conduct a cluster, randomized trial to test the effects of LOCI vs. webinar control condition on full-range leadership and implementation leadership behaviors.
  - H1a: Provider-rated FRL will improve more in the LOCI vs. control condition.
  - H1b: Provider-rated implementation leadership will improve more in the LOCI vs. control condition.
- Aim 2: Test the effect of LOCI on workgroup level implementation climate and psychological safety climate, provider-level attitudes toward MI, and provider implementation citizenship behaviors.
  - H2a: Implementation climate will show greater improvement in LOCI vs. control leader workgroups.
  - H2b: Psychological safety climate will show greater improvement in LOCI vs. control leader workgroups.
  - H2c: Providers in LOCI vs. control condition will report more positive attitudes to MI.
  - H2d: Providers in LOCI vs. control condition will demonstrate greater implementation citizenship.
- Aim 3: Test the effect of LOCI on implementation outcomes MI fidelity and implementation process.
  - H3a: Workgroups in LOCI vs. control condition will show greater improvement in MI fidelity.
  - H3b: Workgroups in LOCI vs. control condition will show greater implementation efficiency as measured by the SIC.
- Aim 4: Explore mediational and cross-level effects (e.g., effects of workgroup level climate on provider attitudes) and test the effects of leadership and strategy on implementation climate, subsequent effects on attitudes toward EBP, and implementation citizenship. Example hypotheses include:
  - H4a: More positive workgroup level implementation climate will be associated with more positive provider-level attitudes toward EBP and implementation citizenship behaviors.
  - H4b: More positive workgroup level psychological safety climate will be associated with more positive provider-level EBP attitudes and implementation citizenship behaviors.
  - H4c: Implementation climate will mediate the effects of leadership on attitudes toward EBP and implementation citizenship behaviors.
  - H4d: Provider attitudes toward EBP and implementation citizenship will mediate the effects of implementation climate on fidelity.

### Design

Multiple study designs were considered by the research team in consultation with collaborating agencies. In order to reduce participant burden, a multiple cohort design, cluster randomized trial that randomizes workgroups within agency to either LOCI or a webinar control was selected. Allocation was determined by the statistician on the project. Three consecutive cohorts of participants will be included. The LOCI intervention lasts for 12 months and the webinar control intervention takes place in months 1, 5, and 8 for each cohort. The unit of analyses will be workgroup, randomized by site within agency with care taken that co-located workgroups are assigned to the same condition to reduce the chances of contamination.

### Setting

The study will take place with workgroups from SUDT agencies in California, USA. California is home to 38 million individuals in 58 counties encompassing urban and vast rural areas. The population is diverse (39.4% non-Hispanic White, 38.2% Hispanic, 14.4% Asian/Pacific Islander, 6.6% Black, 1.7% American Indian). A language other than English is spoken in 38.5% of households. Education levels are 80.8% high school graduate and 30.2% bachelor’s degree or higher, and 14.4% of persons have income below the poverty level.

### Participants

Sample demographics for service providers and workgroup supervisors are expected to be approximately 65% female, 58.7% Caucasian, 27.4% Latino, 18.5% African-American, 2.6% Asian American/Pacific Islander, 2.1% Native American, and 18% “other.” Regarding education, we estimate 18.1% of staff will have a master’s degree, 28.7% college graduates, and 38.8% completed some college level coursework. The proposed settings and providers may also address clients’ co-occurring mental health problems. Consent forms will be available in English and Spanish, and there will also be the capacity for MI coding in both English and Spanish.

#### Recruitment

Executives who have agreed to allow recruitment at their agencies will first identify appropriate workgroups within their agencies (i.e., with opportunity to utilize MI) that will be offered the opportunity to enroll in the study. A workgroup is defined as all direct service providers (e.g., alcohol/drug counselors) who report directly to a single workgroup leader. Once workgroups are identified, executives at each agency will contact eligible workgroup supervisors to invite them to a group phone call with the investigative team to learn more about the study. All eligible participants will be given the opportunity to consent or decline participation in any of the research study components. Participants may cease participation in any part of the research study at any time.

#### Inclusion criteria

Participants for the proposed study will fall into three groups: (1) SUDT service providers (i.e., “service providers” *n* = 360), (2) SUDT workgroup supervisors (i.e., “workgroup supervisors” *n* = 60), and (3) SUDT agency executives and managers (i.e., “executives/managers” *n* = 60), total *N* = 480.

#### Exclusion criteria

Personnel not providing or supervising direct services (e.g., administrative staff) are not included as most of the measures will not be applicable to these staff. Participants must be at least 18 years of age and employed at one of the participating agencies. Supervisors who do not agree to participate in the leadership training (LOCI or webinar), and their staff, will not be eligible to participate.

### LOCI intervention

#### (1) 360° assessment

Consenting workgroup supervisors, service providers who report to them, and executives/managers will participate in the quantitative web-based surveys. A pre-intervention baseline survey precedes intervention deployment for both conditions. All leadership measures will be assessed using a 360° assessment procedure in which leadership ratings are obtained from the leader him/herself, the leader’s subordinates, and the leader’s supervisor.

The 360° assessment data are synthesized into a detailed feedback report and used in the co-creation of a personal leadership development plan for each workgroup leader in the LOCI condition. The research team will only share the feedback reports with each individual LOCI workgroup supervisor. Workgroup supervisors will not be required to share their feedback reports with anyone, including superiors in their organization. Any feedback provided to organization management will utilize aggregate data so that no individual respondents can be identified.

#### (2) Leadership training

##### LOCI intervention

There are three main components of the LOCI intervention: (2.1) training, (2.2) coaching for first-level leaders (workgroup supervisors), and (2.3) tailoring organizational strategies to support the first-level leaders in developing an EBP climate.

2.1 Training

2.1.1 Initial leadership training: The LOCI intervention begins with a 2-day didactic and interactive session. This component includes group introductions, introduction to FRL and implementation leadership, identifying transformational, transactional, and implementation leadership behaviors, identifying behaviors that can be used to build a climate for EBP implementation, and group activities (e.g., breakout groups, interactive exercises, meals) to facilitate social interaction and learning consolidation. The training also addresses implementation climate and the nature of EBPs so that workgroup supervisors learn how to articulate a rationale for how and why EBPs can improve patient and client outcomes. Trainers and coaches work individually with each trainee in a co-creation process in reviewing their 360° assessment data, identifying strengths and areas for development, and setting a timeline for issues to be addressed immediately and those to be addressed later in coaching. Workgroup supervisors emerge with a data-based development plan including broad goals and specific action items that will guide coaching sessions throughout the remainder of the program.

2.1.2 Booster leadership training: Workgroup supervisors in the LOCI condition attend 1-day booster training sessions in 4 and 8 months after the initial training. Prior to each booster session, 360° assessments are completed for updating leadership development plans. Leadership principles, goals, and organizational strategies to support leadership are reinforced through group discussion and problem-solving.

2.1.3 Graduation: Graduation is a rite/ritual deliberately included in LOCI to mark completion of the program. Accomplishments of the participants are celebrated, challenges are processed, and future plans are shared.

2.2 Coaching

Weekly coaching calls are provided for each LOCI supervisor. Coaching calls range from 15–30 min in duration with the goal of keeping participants on track with their goals and development plans, and keeping LOCI principles and strategies in mind. The weekly coaching calls focus on tracking progress in development plans, updating plans based on emergent issues or needs, problem-solving, providing additional leadership support, and identifying organizational strategy needs. Monthly group calls with LOCI leaders are held to facilitate problem-solving and networking among LOCI workgroup supervisors and to discuss their progress and brainstorm solutions to obstacles encountered.

2.3 Organizational strategy meetings (OSMs)

LOCI facilitators meet concurrently with executives, managers, and LOCI workgroup supervisors (within agency) to develop, tailor, and adopt organizational strategies to support the first-level leader in creating an EBP implementation climate in their workgroup. These meetings are held in-person for the first meeting and then by phone for subsequent meetings. As a guiding heuristic, climate-embedding mechanisms (see Additional file 1) are utilized and tailored for each agency and workgroup [51]. The first strategy meeting occurs at each agency site to minimize burden on participants [1]. Follow-up meetings in 4 and 8 months will occur via a web conferencing platform or teleconference. Brief monthly check-in calls (15–30 min) occur with executives only, via teleconference.

##### Webinar condition

The webinar control condition was selected as it is practical and parallels typical time-limited leadership training that is provided for managers and supervisors in public sector service settings. Four webinar sessions will be provided from a well-known leadership training organization. The 1-h webinars focus on leading change and managing work teams. Workgroup supervisors in the webinar control condition will view the webinars at their convenience.

#### (3) MI training

All direct SUDT service providers and workgroup supervisors in both conditions are eligible to receive training in MI. This entails a one-time, 2-day training that occurs approximately 1 week to 1 month after the beginning of the LOCI leadership trainings. The first day addresses basic MI skills, and the second day addresses intermediate MI skills. MI training typically includes didactic training on topics such as the spirit and principles of MI and interactive training and interactive exercises in order to provide skill building and practice. MI trainers are Motivational Interviewing Network of Trainers (MINT) members.

#### (4) MI fidelity data collection

Participants will be asked to audio record and upload all of their sessions with clients where MI could be utilized. A digital audio recorder will be provided for each direct service provider. Although clients receiving the MI intervention may be heard on the audio recordings of these MI sessions, clients themselves will not be participants in the study. The research team will receive no identifying information about clients, and although no identifiable client information will be collected, informed consent will be sought from clients stating that they will allow the audio recording of their sessions. After audio recording MI sessions, participating service providers will log into a secure, HIPAA-compliant file-sharing website to upload each audio file. One randomly selected recording per counselor per month will be coded for fidelity, and a fidelity report for each provider will be provided back to the supervisor. Supervisors have the additional option of receiving individual training on providing fidelity feedback and coaching for direct service staff.

### Data collection and management

Data are to be collected at baseline (prior to the interventions), 4, 8, 12, and 16 months (4 months after the end of the LOCI intervention). Organizational data will be collected from all participants (service providers, workgroup supervisors, and executives/mid-managers) via web surveys and downloaded into a database programmed for error identification and checking. Each type of respondent will complete a set of measures in their survey, and not all measures will be collected at each time point. The provider/supervisor surveys will take about 20–30 min to complete, whereas the survey for executives/mid-managers will take about 5–10 minutes per supervisee. Participants will be compensated with a $25 electronic gift card via email for completing each survey. A portion of the data collected in these surveys (i.e., FRL, implementation leadership, EBPAS, EBP implementation climate) will be shared with supervisors in the LOCI condition as a part of the leadership development intervention. Data from managers/executives will be shared with the supervisors, and this is comparable to a typical performance review. Data from service providers will be shared with their supervisors in an aggregate manner to protect the confidentiality of individual provider responses. All data will be stored on a secure server with incremental and weekly full back up.

### Measures

#### Demographics

Data to be collected includes age, sex, education level, professional status (e.g., intern vs. professional), and job tenure [13].

#### Full-range leadership

The Multifactor Leadership Questionnaire (MLQ) [70] assesses transformational and transactional leadership. MLQ scores are associated with organizational climate and working alliance in behavioral health agencies [71] and predict organizational effectiveness. Service providers rate the extent to which their immediate supervisor engages in specific behaviors measured by the MLQ, and executives do the same for their supervisees. Each behavior is rated on a 5-point scale (0 = Not at all, 4 = Frequently, if not always). The MLQ has good to excellent psychometric properties with Cronbach’s alphas ranging from .76 to .90. Transformational leadership is assessed by four subscales: Idealized Influence (8 items, *α* = .87), Inspirational Motivation (4 items, *α* = .91), Intellectual Stimulation (4 items, *α* = .90), and Individual Consideration (4 items, *α* = .90). Transactional leadership is assessed with two subscales of Contingent Reward (4 items, *α* = .87) and Active Management-by-Exception (4 items, *α* = .74).

#### Implementation leadership

The Implementation Leadership Scale (ILS) is a brief measure of unit level leadership for EBP implementation with excellent reliability and convergent and discriminant validity [23]. The four ILS subscales are Proactive Leadership (*α* = .95), Knowledgeable Leadership (*α* = .96), Supportive Leadership (*α* = .95), and Perseverant Leadership (*α* = .96), and the total score is *α* = .98. Each item is scored on a 5-point Likert-type scale (0 = Not at all, 4 = To a very great extent).

#### Implementation climate

The Implementation Climate Scale (ICS) [30] was adapted from Klein and colleagues’ study of innovation implementation [31] and considering Schein’s construct of “embedding mechanisms” [2]. The ICS assesses employees’ shared perceptions of the policies, practices, procedures, and behaviors that are expected, supported, and rewarded to facilitate effective EBP implementation. All items are scored on a 5-point Likert-type scale (0 = Not at all to 4 = To a very great extent). The ICS has excellent internal consistency and convergent and discriminant validity. The ICS has an overall Cronbach’s alpha of .91 (18 items, 3 items on each subscale). The six subscales are Focus on EBP (*α* = .91), Educational Support for EBP (*α* = .84), Recognition for EBP (*α* = .88), Rewards for EBP (*α* = .81), Selection for EBP (*α* = .89), and Selection for Openness (*α* = .91).

#### Psychological safety climate

Psychological safety climate (PSC) will be assessed with the PSC scale [72], which assesses employees’ shared perceptions of organizational policies, procedures, and behaviors regarding a supportive, safe work environment for taking interpersonal risks. The PSC has seven items scaled from 0 (Doesn’t apply at all) to 4 (Entirely applies) with good internal consistency reliability (*α* = .82), construct, and concurrent validity. PSC scores are associated with workgroup membership, contextual support, and interactions with the workgroup supervisor.

#### Attitudes toward EBP

The Evidence-Based Practice Attitude Scale (EBPAS) [73] will assess individuals’ attitudes toward EBP. The EBPAS has 15 items with four subscales that assess attitudes toward adoption of EBP as a function of perceived *Appeal* of EBP, *Requirements* to use EBP, provider *Openness*, and perceived *Divergence* between EBP and usual care. EBPAS total scores (*α* = .76) represent global attitudes toward adoption of EBP and subscale alphas range from .66 to .91. EBPAS responses are scored on a 5-point scale (0 = Not at all, 4 = To a very great extent), and scores are associated with individual provider-level attributes and organizational characteristics [73–75].

#### EBP Implementation Citizenship Behavior

The Implementation Citizenship Behavior Scale (ICBS) [69] will assess individual EBP implementation citizenship. The ICBS was adapted from an existing measure of safety citizenship in the workplace [76] and assesses the extent to which individual workgroup members go beyond minimum requirements to support successful EBP implementation in regard to helping others and keeping informed. The ICBS is comprised of 6 items that are scored on a 5-point scale (0 = Not at all, 4 = Frequently, if not always). The ICBS has demonstrated excellent internal consistency reliability for the Helping (*α* = .93, 3 items), Keeping Informed (*α* = .91, 3 items), and ICBS Total EBP Citizenship scales (*α* = .93).

#### Assessment of Prior MI Use

The Assessment of Prior MI Use measure was developed for this study to measure the extent to which service providers have been exposed to MI (i.e., previoustraining and use of MI with clients) prior to the MI training offered as a part of the study. All participants will be asked about prior training and current use of MI, in addition to study data on number of recordings and session uploads, to aid in determining penetration at provider and client levels. Those with prior experience are asked to estimate the percentage of clients with whom they use MI and the extent to which they use MI with fidelity. These data can be used as provider-level covariates in quantitative analyses.

#### LOCI Component Assessment

The LOCI Component Assessment was developed to increase understanding of the relative importance and utility of each of the six components of LOCI. It measures the extent to which LOCI trainees perceive each component to be important and useful in achieving change in leadership and improving EBP implementation.

#### MI Coach Rating Scale

The MI Coaching Rating Scale (MI-CRS) [77] will be used to assess fidelity. This scale was developed based on the MI Treatment Integrity Instrument (MITI) [78] that has demonstrated inter-rater reliability and differentiates between MI and usual care [79]. The MI-CRS was developed utilizing Item Response Theory [80, 81] and is designed to be useful for research and practical for agencies to utilize for their own in-house fidelity monitoring. Five items address the relational components of MI, and 5 items address the technical components of MI, with each item scored on a 4-point scale (1 = Poor, 2 = Fair, 3 = Good, 4 = Excellent). A mean of 3.5 and above on a given component is considered solid competence, a mean of 2.5 to 3.5 is considered beginner competence, and a mean score of less than 2.5 is considered below competence. Component scores will be averaged to compute a total mean fidelity score. Research staff will use the MI-CRS to code audio recordings of client sessions in which MI is used.

#### Stages of Implementation Completion

The stages of implementation completion (SIC) measure will be used to assess implementation process [82]. It assesses activities that occur during eight stages of the measure, which fit within the EPIS implementation phases of Preparation (e.g., SIC pre-implementation) and Implementation. The SIC will be adapted for MI and LOCI to assess each workgroup’s progress toward successful MI implementation in consultation with the measure developer, and completed by the investigative team based on ongoing documentation of the dates at which each SIC activity and stage is completed. Three scores are calculated: (1) a duration score (i.e., time in days that a site takes in a stage), (2) proportion score (i.e., percentage of activities completed within a stage), and (3) the SIC stage score (i.e., number of stages completed). The SIC has demonstrated construct and predictive validity and identifies and predicts variation in implementation process [83, 84].

#### Assessment of Climate Embedding Mechanisms (ACEM)

The ACEM was developed for this study and will be used to measure the organizational strategies developed and used by each organization and leader trainee. The first part of the measure includes qualitative items to identify all strategies that are being used within a workgroup, the larger organization, and across levels to embed a climate for the implementation of MI. For each strategy identified, quantitative items measure each strategy’s frequency of use and level of emphasis. The result is a list of strategies used within each workgroup and organization to support the implementation of MI.

#### LOCI Feasibility, Acceptability, Utility

The LOCI Feasibility, Acceptability, Utility scale [1] is an 11-item measure developed for the LOCI pilot study to assess the LOCI training.

### Data analyses

The study will obtain estimates of the leadership intervention effect by comparing multiple intervention measures on provider-rated leadership, workplace climate, work attitudes, and attitudes toward adopting and implementing EBPs. The workgroup will be the unit of analysis, with sites randomized within agency to ensure that the 5% of co-located workgroups will be randomized into the same condition to avoid contamination. For 95% of workgroups, workgroup is synonymous with site. We will stratify by the number of workgroups per site to ensure equal workgroups in each condition.

Data analytic strategies will follow the recommendations of Brown et al. [85] and the Prevention Science and Methodology Group for randomized field trials [86]. Preliminary data screening and cleaning will require examination of data distributions for normality and missing data patterns at both the univariate and multivariate levels. Once complete, hierarchical linear modeling (HLM) [87] will be used as the primary statistical model due to the nested structure of the data: repeated measures (time) (level-1) nested within service providers (level-2) nested within workgroups (level 3). Because randomization is at the workgroup level, and due to the statistical complications associated with the small number of agencies and a number of workgroups being located at the same site, dummy-coded agency and site variables will be entered at the level of the workgroups for all analyses, which implicitly controls for agency and site effects [88]. Random effect variance will be evaluated for the remaining levels of the nested data structure. In the presence of near zero estimates of variance estimates, terms will be removed from the statistical model.

HLM analyses for aims 1 and 2 will test specific intervention effects (LOCI vs. webinar control condition) on target outcomes (e.g., leadership behaviors, implementation climate). Of primary interest in these analyses is the Time (level-1) × Intervention Status (level-3) cross-level interaction effect. If the cross-level interaction term(s) are statistically significant, follow-up examination of these effects will follow the procedures of Preacher, Curran, and Bauer [89]. Implementation of cross-classified models will be explored given that some shifting or personnel may occur during the course of the study [90].

Aim 3 will test fidelity. The statistical methods used will be based on the HLM models described for the first two aims; however, since measurements for this outcome are averaged over time, there will not be a modeled covariance structure. For count outcomes related to implementation process (workgroup level), we will use a negative binomial regression model instead of a Poisson regression model to allow for the possibility of over-dispersion.

Finally, mediational effects described in aim 4 will use procedures for multilevel data structures outlined in MacKinnon [91]. For all analyses, covariates will be considered at both the provider and workgroup level. Inclusion of confounder variables at the provider level is important given that randomization to LOCI vs. control condition will not occur at this level. Inclusion of covariates at the workgroup level will adjust for chance imbalances and increase the precision of the analyses.

## Discussion

LOCI has been developed to be feasible and effective for organizations to create a positive climate and fertile context for EBP implementation. The approach seeks to cultivate and sustain both effective general and implementation leadership in conjunction with organizational strategies and supports that will remain after the implementation strategy has ended. LOCI’s strategic in-person training combined with brief weekly coaching minimizes burden and promotes ongoing cognitive processing (i.e., being mindful of leadership and implementation issues) and enables ongoing and repeated efforts at behavior change. Effective leadership in health and allied health services is associated with more positive staff attitudes toward adopting EBP, provider adoption of EBPs, improved staff work attitudes, and performance [51]. Effective leadership is also critical in the successful and sustainable implementation of innovation [31, 92]. LOCI builds on these findings to garner broader organizational stuructures and processes to support implementation and sustainment.

Most agencies already provide manager trainings, but these tend to be more superficial and lack follow-up to support leader and organizational behavior change. There are many off-the-shelf leadership development programs, but such programs may lack utility because (1) they tend to be broad in scope and not designed to develop strategic climates for EBP, (2) are often not based on empirically supported approaches and curricula, (3) are often time-limited with little or no follow-up and inconsistent with learning theory that suggests interventions distributed over time and coupled with coaching and practice are more effective [93], and (4) are rarely tested for evidence of effectiveness or practical utility. The work proposed in this study addresses each of these limitations by (1) focusing on developing strategic climate for EBP in SUDT services, (2) utilizing an empirically based curriculum with measurable outcomes [94], (3) combining didactic and interactive training with ongoing coaching to support learning and behavior change, (4) linking first-level leader development with targeted organizational support strategies, and (5) empirically testing LOCI to determine its effects on proximal (leadership behaviors, implementation climate) and distal (provider attitudes and behaviors, fidelity, implementation process) outcomes.

While many SUDT providers are trained in MI, few deliver MI with fidelity. This study addresses a major gap in the way in which EBPs are “implemented” in usual care SUDT settings. It is not enough to “train and hope” that EBPs will be delivered with fidelity and in a manner that will lead to improved patient outcomes. What is needed are more comprehensive approaches to changing the context of community-based services to be ready, willing, and able to implement appropriate evidence-based service models to improve patient outcomes. The LOCI intervention holds promise in regard to these goals.

## Additional files

Additional file 1: Climate Embedding Mechanisms. (PDF 242 kb)
