# Supplementary material for: Testing the leadership and organizational change for implementation (LOCI) intervention in substance abuse treatment: a cluster randomized trial study protocol
Source: Implement Sci. 2017 Mar 3;12:29. doi: 10.1186/s13012-017-0562-3 (PMC5335741; doi:10.1186/s13012-017-0562-3)
Supplement: Additional file 1: — Climate Embedding Mechanisms. (PDF 242 kb) [file 13012_2017_562_MOESM1_ESM.pdf]

Additional File 1

EMBEDDING MECHANISMS

The six primary embedding mechanisms described by Schein (2010) are as follows:

1. what leaders pay attention to, measure, and control on a regular basis;
2. how leaders react to critical incidents and organizational crises;
3. how leaders allocate resources;
4. deliberate role modeling, teaching, and coaching;
5. how leaders allocate rewards and status; and
6. how leaders recruit, select, promote, and excommunicate.

Schein also outlined secondary articulation and reinforcement mechanisms that support and perpetuate the organization’s climate, provided they are consistent with the primary mechanisms above. The six secondary mechanisms are as follows:

1. organizational design and structure
2. organizational systems and procedures
3. rites and rituals of the organization
4. design of physical space, facades, and buildings
5. stories about important events and people
6. formal statements of organizational philosophy, creeds, and charters

Schein E. 2010. *Organizational Culture and Leadership*. San Francisco: Wiley
